# Gender bias in clinicians’ pathologization of atypical sexuality: a randomized controlled trial with mental health professionals

**DOI:** 10.1038/s41598-018-22108-z

**Published:** 2018-02-27

**Authors:** Johannes Fuss, Peer Briken, Verena Klein

**Affiliations:** Institute for Sex Research and Forensic Psychiatry, Center of Psychosocial Medicine, University Medical Center Hamburg-Eppendorf, Hamburg, Germany

## Abstract

The psychiatric classification of “normal” versus disordered sexual behavior has been a subject of some dispute. Although atypical sexual interests have been viewed traditionally as typically male, epidemiological data indicate its presence in both genders. We examined how gender and sexual orientation influence whether or not atypical sexual behavior is classified as a mental disorder. Mental health professionals (N = 546) were presented with five case vignettes where subjects exhibit paraphilic behaviors; one case with psychotic symptoms served as the control condition. For each vignette we randomly changed the described subject’s gender (male/female), sexual orientation (homosexual/heterosexual), and presented diagnostic criteria (fulfilled/ambiguous). Female subjects were significantly less pathologized and overall less stigmatized in terms of exhibitionistic, frotteuristic, sexual sadistic and pedophilic behavior. On the other hand, female sexual behavior that fulfilled diagnostic criteria for masochistic disorder was more pathologized. Our results demonstrate that nosologically irrelevant factors, which may be related to different sexual norms for men and women, affect clinicians’ decisions regarding atypical sexuality.

## Introduction

Historically, the distinction between “normal” and atypical sexual behaviors has undergone profound changes, because it is based on a socially-constructed sexual morality that is ever changing^1^. Since Krafft-Ebing’s *Psychopathia sexualis*^2^ in 1886, it has increasingly become the task of psychiatry to define pathological forms of sexual behavior. Since that time, historical changes in social norms have found their expression in new terminologies and diagnostic criteria for sexual behaviors across time^3,4^. Sexual practices such as masturbation were once considered as diseases^5,6^ and, up until 1973, the Diagnostic Manual of Mental Disorders (DSM)-II pathologized homosexuality as a sexual deviation^7^. Psychiatric nosology has also shaped such female sexual pathologies as frigidity, hysteria and nymphomania^8,9^. Additionally, the influence of psychiatric diagnoses on criminal law highlights its impact on society^10^.

Current research indicates that atypical sexual fantasies are much more common in the general population than the terms “deviant” or “atypical” might suggest^11–16^. Importantly, most people with atypical sexual fantasies or behaviors do not fulfill the criteria for a mental disorder^17^. Recently, this has been underlined by the latest update of the DSM. The DSM-5 makes a distinction between atypical sexual behaviors - paraphilias - and paraphilic disorders. Only if a paraphilia causes distress or harm to oneself or to others, such as acting a behavior upon a person without consent, can a paraphilic disorder be diagnosed. Thus, atypical sexual behaviors are not ipso facto mental disorders. With a different terminology, this has also been true for the fourth edition of the DSM^18^.

Even though the DSM-5 update was applauded by some researchers and clinicians, others have criticized the concept of paraphilias and paraphilic disorders in general. Most predominantly discussed are the vague and problematic boundaries between normal and pathological behaviors, the lack of scientific evidence for this distinction, and the possible legal consequences in criminal trials^19–22^. Lately, some researchers have therefore argued for the deletion of paraphilias from the DSM, especially those behaviors that are performed solitarily or consensually^19^. Most authors nowadays agree with the removal of legal and consenting paraphilias, as proposed for the forthcoming eleventh revision of the International Classification of Diseases and Related Health Problems (ICD-11)^23^. In line, paraphilias were among the most frequently-recommended diagnoses for removal from mental disorders classification by mental health professionals (MHP)^24^ and some Scandinavian countries have already removed certain paraphilias from their national psychiatric manuals^25^. In contrast, in other countries, practitioners are still in danger of prosecution^26^ and recently a U.S. Court decided that engaging in consensual BDSM sexual activity is not a constitutional right^27^.

In general, men seem to be more likely to report greater interest in paraphilic interests and behaviors than women^17^, even if recent evidence challenges this assumption^11,12^. However, paraphilias are *perceived* as typical male mental disorders, and are highly stigmatizing compared to other mental disorders^28^. Various editions of the DSM and ICD illustrate that “…kinky behaviors are perceived as an attribute or tendency of men”^4^. The DSM-IV concluded that paraphilias “…are almost never diagnosed in females”. The question as to whether this conclusion is an expression of a male construction of paraphilias, or simply represents the clinical reality, remains yet unclear. Male gender is, however, not a diagnostic criterion for paraphilic disorders, and although the prevalence for paraphilic disorders seems to be higher in men, robust epidemiological data is sparse^17,29^.

The diagnostic criteria for paraphilias in DSM and ICD are nowadays designed for use as an objective tool to differentiate between non-disordered and mentally-disordered sexuality. Such criteria are intended to help reduce the influence of a psychiatrist’s cultural attitudes and her or his own socialization when making a diagnosis. Consequently, the same behaviors in different people should be diagnosed comparably. Non-decisive factors such as the gender or sexual orientation of the patient should not affect the likelihood of diagnosing a mental disorder, especially because biased clinical decisions might have harmful consequences associated with negative healthcare outcomes^30^.

The aim of our present study was to investigate for the first time how gender and sexual orientation affect the diagnosis and stigmatization of atypical sexual behaviors among MHP. To account for the multifaceted nature of stigma^31^, different stigma dimensions (desire for social distance, blame, and perceived dangerousness) were assessed. Based on empirical evidence suggesting that atypical sexual behaviors are predominantly perceived as masculine, we hypothesized that men and women with atypical sexuality would be evaluated differently.

## Results

We provided mental health professionals (N = 546, Table 1) with case vignettes of different paraphilic behaviors and a control vignette, and randomly changed the gender and sexual orientation of the described subject. We also randomly changed the severity of the described symptoms to include a condition that should affect the evaluation based on the diagnostic criteria. Thus, MHP were randomly assigned to one cell of a 2 “gender” (male/female) × 2 “sexual orientation” (homosexual/heterosexual) × 2 “diagnostic criteria” (fulfilled/ambiguous) factorial design. They were asked to evaluate the cases, and to estimate whether or not a mental disorder is present, indicate its biological or psychological underpinnings, and provide various measures of stigma. Sixty-four % women and 35% men participated in the present study. Most participants were psychologists (59%) or psychiatrists (36%) who had, on average, 11 years of clinical experience (further details see Table 1).

**Table 1:** Sociodemographic variables of participating mental health professionals.

*Participating Mental Health professionals* [*n*]	546
*Sex* [*n*]	
Female	347 (63.6%)
Male	189 (34.6%)
No answer	10 (1.8%)
Age [Mean in years ± SEM]	40 ± 0.5
Clinical experience [Mean in years ± SEM]	11 ± 0.4
*Profession* [*n*]	
Psychologist	323 (59.2%)
Psychiatrist	198 (36.3%)
Other	15 (2.7%)
No answer	10 (1.8%)
*Additional qualification* [*n*]	
No additional qualification	454 (83.2%)
Forensic psychiatry	53 (9.7%)
Sexual medicine	23 (4.2%)
No answer	16 (2.9%)
*Country* [*n*]	
Germany	494 (90.5%)
Austria	30 (5.5%)
German-speaking Switzerland	8 (1.5%)
No answer	14 (2.5%)
*In Relationship* [*n*]	
Yes	444 (81.3%)
No	91 (16.7%)
No answer	11 (2%)
*Sexual orientation* [*n*]	
Heterosexual	480 (87.9%)
Homosexual	31 (5.7%)
Bisexual	23 (4.2%)
No answer	12 (2.1%)
*Method of psychotherapy* [*n*]	
Cognitive-behavioral therapy	247 (45.2%)
Psychodynamic psychotherapy	159 (29.1%)
Psychoanalysis	50 (9.2%)
Other	24 (4.4%)
Systemic therapy	10 (1.8%)
Gestalt therapy	3 (0.5%)
No answer	53 (9.7%)
*Religion* [*n*]	
Christianity	302 (55.3%)
No religion	207 (37.9%)
Buddhism	21 (3.8%)
Islam	6 (1.1%)
Judaism	3 (0.5%)
Other religion	3 (0.5%)
Hinduism	2 (0.4%)
No answer	2 (0.4%)
Political attitude [6-point Likert scale from 1 *liberal* to 6 *conservative*]	2.4 ± 0.05
*Attitudes [6-point Likert scale from 1 strongly disagree to 6 strongly agree]*	
Sexual difference between men and women	3.8 ± 0.05
I am religious	2.7 ± 0.07
I am a member of a minority	2.0 ± 0.05

The first vignette described an individual with psychotic symptoms followed by five vignettes describing subjects with paraphilic sexual interests or behaviors (exhibitionistic, frotteuristic, sexual sadistic, pedophilic, sexual masochistic) (for detailed descriptions of the vignettes see Supplementary File 1).

**Figure 1:**
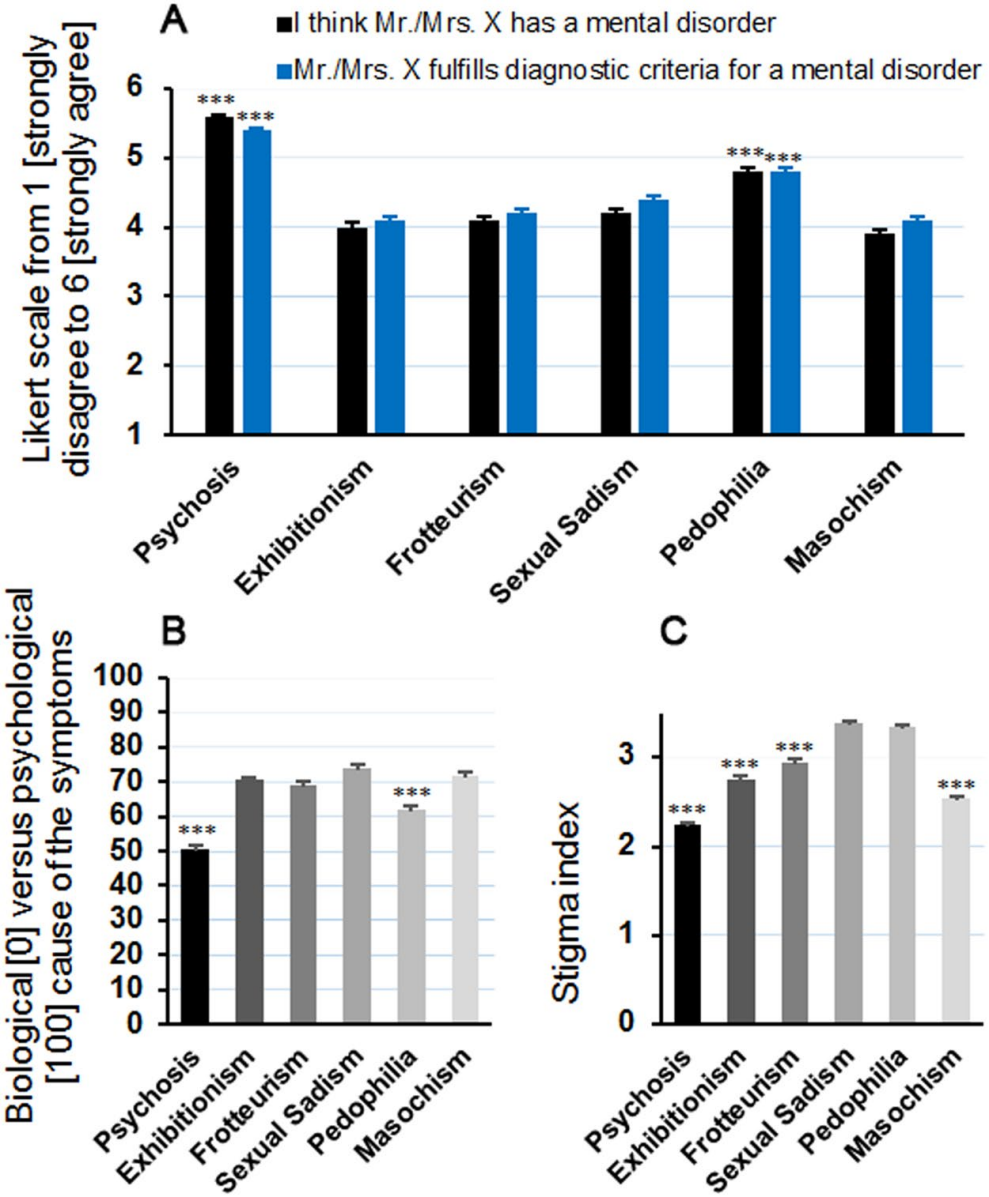
Mental health professionals responded differently to the six vignettes. While psychotic behavior were rated highest regarding psychopathology (**A**) they were also the least stigmatized (**C**) and the presumed psychological component was lowest (**B**). Pedophilic behavior were rated to be significantly more biologically-based and mentally-disordered compared to all other paraphilic behavior. Pedophilic and sexual sadistic behavior had the highest stigma index. Sexual masochism was significantly less stigmatized compared to all other paraphilias. ***Indicates a significant difference in post-hoc analysis compared to all other vignettes.

Estimation of psychopathology was lower (p < 0.001) in all of the paraphilic vignettes compared to the psychosis vignette (Fig. 1) with regard to the question as to whether or not the subject is mentally disordered (F_5,3222_ = 162.95, p < 0.001) and fulfills the criteria for a mental disorder (F_5,3182_ = 93.21, p < 0.001). On average, the mean scores of all the vignettes were >3.5 (on a rating between strongly disagree “1” and strongly agree “6”) and thus leaning toward the pathological end of the scale. The pedophilic vignette was significantly more pathologized compared to the other paraphilic vignettes (p < 0.001). When participants had to indicate “the cause” of the symptoms, it was also found that a significant difference existed between vignettes (F_5,3270_ = 61.91, p < 0.001). While psychotic symptoms were comparably attributed to both biological and psychological factors (49.5% vs. 50.5%), the paraphilic vignettes were significantly more often ascribed to psychological factors (~70%, p < 0.001). The pedophilic vignette (62 ± 1% psychological factors) was again somewhat between the psychotic vignette and the other paraphilias. Pedophilia was thus estimated of being remarkably more “biological” and “mentally disordered” than the other paraphilias (p < 0.001), especially in terms of the full criteria condition (Suppl. Figure 1). Overall, the behaviors presented also differed in the degree to which they were stigmatized (F_5,3183_ = 202.09, p < 0.001). A Bonferroni post-hoc analysis indicated that the overall stigmatization scores were lowest in the psychosis condition, followed by masochism, exhibitionism, and frotteurism, and highest in the pedophilia and sexual sadism condition (Fig. 1C).

Both questions addressing how participants evaluate the mental health of the subject correlated highly (Fig. 1; for example, R = 0.77, p < 0.001 for the psychosis vignette and R = 0.81, p < 0.001 for the exhibitionism vignette). We calculated a “psychopathology factor” as the mean of both values for further analysis in order to include both a subjective and an objective estimation of mental disorder. This psychopathology factor was included in a 2 × 2 × 2 ANOVA. In this 3-factorial ANOVA, the ambiguous condition was estimated as less pathological in all of the vignettes (psychosis: F_1,513_ = 9.78, p = 0.002, exhibitionism: F_1,510_ = 56.14, p < 0.001, frotteurism: F_1,516_ = 20.85, p < 0.001, sexual sadism: F_1,513_ = 14.79, p < 0.001, pedophilia: F_1,538_ = 105.53, p < 0.001, and sexual masochism: F_1,519_ = 8.85, p = 0.003). Thus, we confirmed that the manipulation of the vignettes affected the participants’ estimation of psychopathology.

**Figure 2:**
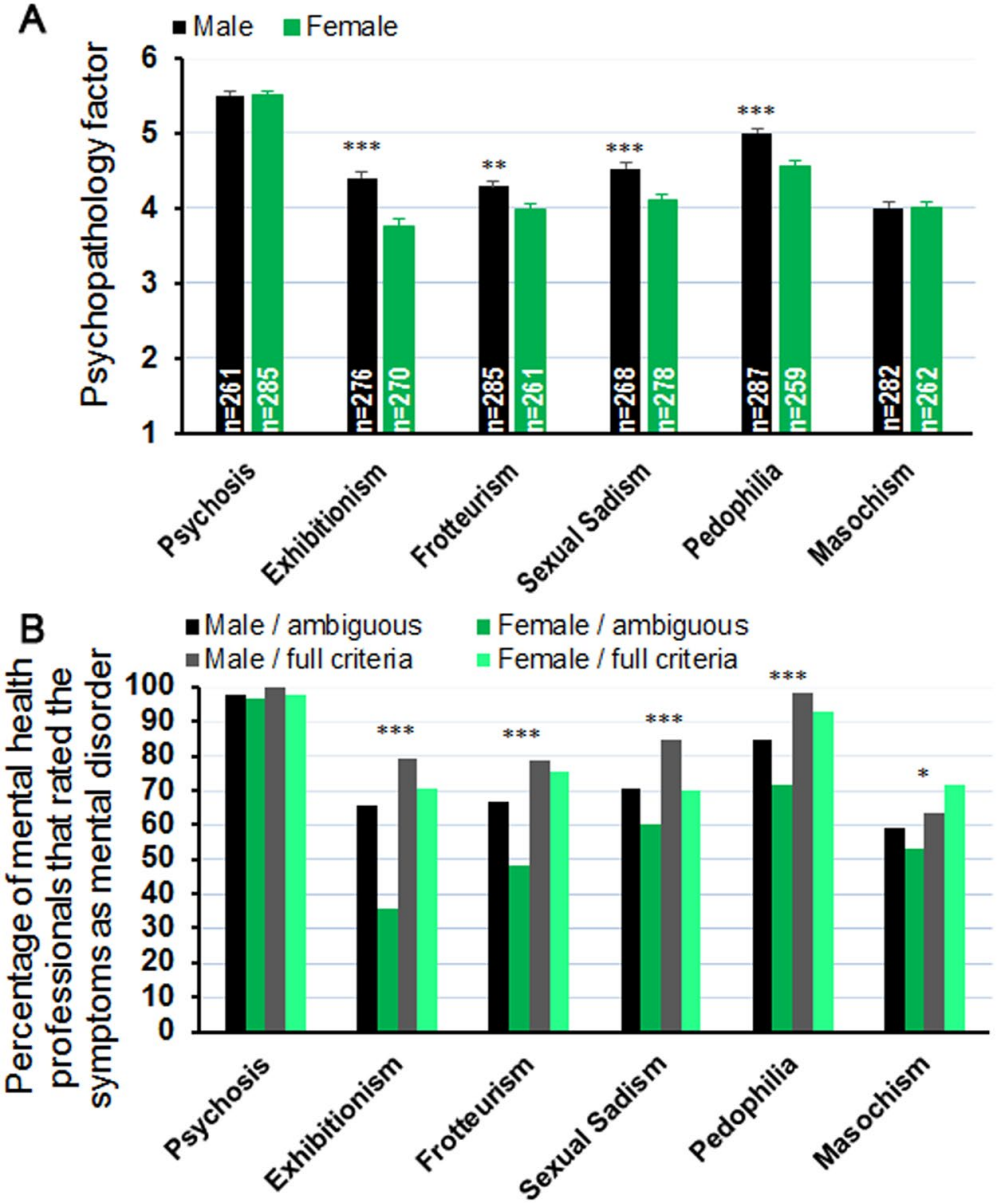
Female subjects were less pathologized in the exhibitionistic, frotteuristic, sexual sadistic and pedophilic vignette (**A**). In line with this, the percentage of mental health professionals who rated the symptoms as a mental disorder (=psychopathology factor >3.5) was lower in the female condition in these vignettes (**B**). In contrast, the psychopathology factor was comparable between males and females in the masochistic vignette (**A**). Interestingly, while females were also less pathologized in the ambiguous condition with the number of females who received a mental disorder diagnosis increased in the full criteria condition (**B**). Neither the psychopathology factor, nor the percentage of mental health professionals that gave a diagnosis was affected by gender in the psychosis control condition. *,**,***Indicate a significant difference in post-hoc analysis compared to all other vignettes with p < 0.05, 0.01, 0.001, respectively.

Interestingly, MHP also gave lower psychopathology factors to female subjects in most of the paraphilic vignettes (exhibitionism: F_1,510_ = 35.95, p < 0.001, frotteurism: F_1,516_ = 7.34, p = 0.007, sexual sadism: F_1,513_ = 17.46, p < 0.001, and pedophilia: F_1,523_ = 26.50, p < 0.001, Fig. 2A). In contrast, with regard to the control and sexual masochism vignettes, gender did not influence the participants’ estimation of psychopathology (psychosis: F_1,513_ = 0.01, p = 0.917, sexual masochism: F_1,519_ = 0.02, p = 0.964). The percentage of MHP who rated the symptoms described as a mental disorder (psychopathology factor >3.5) also differed profoundly from vignette to vignette (Fig. 2B). Almost 100% of the participants judged the psychotic symptoms as indicating a mental disorder, independent of gender or criteria (χ²(3) = 3.43, p = 0.329, Fig. 2B). In contrast, in the exhibitionistic (χ²(3) = 62.20, p < 0.001), frotteuristic (χ²(3) = 34.14, p < 0.001), sexual sadistic (χ²(3) = 21.03, p < 0.001) and pedophilic (χ²(3) = 47.12, p < 0.001) vignettes, female subjects were less likely to be diagnosed as mentally disordered. This gender bias was particularly pronounced in the ambiguous condition (Fig. 2B). For example, while 65% of MHP pathologized an exhibitionistic man, only 36% pathologized the female subject in the ambiguous condition. Interestingly, we also found a gender effect for the sexual masochism vignette (χ²(3) = 10.23, p < 0.017). In the ambiguous condition, females were less likely to be pathologized. In contrast, when full diagnostic criteria were present, the percentage of MHP who diagnosed a mental disorder was almost 10% higher in the female condition.

In terms of different stigma dimensions, a gender difference occurred for all paraphilias – with the exception of masochism – on the dimensions desire for social distance and perceived dangerousness. Male subjects were perceived as being more dangerous and provoked more desire for social distance compared to female subjects. In addition, MHP blamed male subjects more than female subjects for frotteuristic behaviors (see Table 2).

**Table 2:** Stigmatization of different paraphilic behaviors by MHP.

Stigma outcomes:	Desire for Social Distance	Blame	Perceived Dangerousness
*Psychosis*			
Female (Mean ± SEM)	6.20 ± 0.11	5.14 ± 0.11	3.84 ± 0.08
Male (Mean ± SEM)	6.61 ± 0.11	5.45 ± 0.12	4.43 ± 0.09
F-value	7.13	3.59	24.55
p-value	0.01	0.06	<0.001
*Exhibitionism*			
Female (Mean ± SEM)	6.45 ± 0.13	8.34 ± 0.14	3.00 ± 0.10
Male (Mean ± SEM)	7.74 ± 0.13	8.70 ± 0.14	4.55 ± 0.10
F-value	48.98	3.18	132.43
p-value	<0.001	0.08	<0.001
*Frotteurism*			
Female (Mean ± SEM)	6.20 ± 0.14	8.96 ± 0.16	3.67 ± 0.13
Male (Mean ± SEM)	7.37 ± 0.13	9.73 ± 0.15	5.31 ± 0.12
F-value	37.52	12.29	87.10
p-value	<0.001	<0.001	<0.001
*Sadism*			
Female (Mean ± SEM)	6.67 ± 0.14	10.40 ± 0.16	5.65 ± 0.15
Male (Mean ± SEM)	7.63 ± 0.15	10.45 ± 0.16	6.67 ± 0.15
F-value	21.90	0.04	22.94
p-value	<0.001	0.85	<0.001
*Pedophilia*			
Female (Mean ± SEM)	7.55 ± 0.14	9.01 ± 0.16	5.87 ± 0.14
Male (Mean ± SEM)	8.55 ± 0.15	9.17 ± 0.15	6.66 ± 0.13
F-value	24.45	0.52	17.09
p-value	<0.001	0.47	<0.001
*Masochism*			
Female (Mean ± SEM)	6.21 ± 0.14	8.42 ± 0.14	3.37 ± 0.10
Male (Mean ± SEM)	6.08 ± 0.14	8.23 ± 0.13	3.33 ± 0.10
F-value	0.40	1.03	0.04
p-value	0.53	0.31	0.84

**Figure 3:**
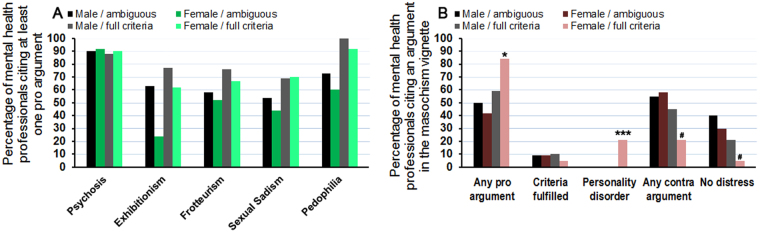
Percentage of mental health professionals listing at least one pro argument for a mental disorder in their opinions in the open text box (**A**). Especially in the ambiguous condition, the percentage of mental health professionals in the female condition was lower. Again, sexual masochism was differently evaluated in the ambiguous and full criteria condition (**B**). The percentage of mental health professionals listing at least one pro argument was increased in the full criteria condition and reduced in the ambiguous condition. Moreover, more than 20% of mental health professionals diagnosed a personality disorder for the female subject in the full criteria condition. *,***Indicate a significant difference in post-hoc analysis compared to all other vignettes with p < 0.05, 0.001, respectively. ^#^Indicates a difference on trend level p < 0.1.

Roughly 20–25% of all participants provided explanations concerning their diagnoses in the open-ended text boxes. These qualitative data were rated by two independent raters and sorted into different coding categories (displayed in Supplemental Table 1). In line with the quantitative results, under ambiguous conditions, MHP listed on average less pro and more contra arguments compared to the full criteria condition (Fig. 3A). In the female compared to the male condition, the percentage of MHP who listed at least one pro argument was lower in the exhibitionism, frotteurism, sexual sadism and pedophilia vignettes. This was more pronounced under ambiguous conditions (Fig. 3A). In contrast, in terms of sexual masochism in the full criteria condition, the percentage who listed at least one pro argument was 25% higher in the female condition (χ²(3) = 8.99, p = 0.029, Fig. 3B). Intriguingly, personality disorders were only listed as a reason for pathologizing atypical sexual behavior in the female/full criteria condition by 21% of the participants (χ²(3) = 17.98, p < 0.001, Fig. 3B). Moreover, less contra arguments (21%) were to be found in the female/full criteria condition, in contrast to 45–58% in the other three conditions (χ²(3) = 7.19, p = 0.066, Fig. 3B).

In all cases, the sexual orientation of the subject did not affect the psychopathology factor in the present study. Moreover, the gender of the participating MHP had no effect on the results – pathologization of male paraphilic behaviors was more pronounced in both male and female MHP. Additionally, mental health professionals tended to pathologize male paraphilic behaviors to a higher extent, independently of their therapeutic background. Only for the sadism vignette did we find an effect of the therapeutic background (F_2,391_ = 5.32, p = 0.005). MHP trained in psychoanalysis pathologized subjects expressing sexual sadistic behaviors to a greater extent than MHP trained in cognitive-behavioral therapy (p = 0.01).

## Discussion

The present study demonstrates for the first time how a person’s gender can influence their likelihood of being pathologized with a paraphilic disorder. One possible reason may be that MHP tend to ignore or downplay paraphilic interests in women. Indeed, paraphilias seem to be particularly perceived (and possibly overestimated) as masculine mental disorders^28^. In contrast, seen from a historical perspective, women’s sexuality was more socially sanctioned than male sexuality, and indeed women’s sexual desire was once deemed as socially disruptive^8,32^. Before Krafft-Ebing’s *Psychopathia sexualis*, sexual deviancy was even considered a sign of feminization in men^33^. Our data indicate different contemporary sexual norms for women and men, which impact the evaluation of atypical sexual behavior within the mental health system. Future research should address to what extent this result is associated with sociocultural norms, and the social construction of female sexuality as being passive and submissive, and male sexuality as being dominant and aggressive^34^. However, the result that female subjects were more likely to be pathologized in the full criteria masochism condition points to activated gender stereotypes in the clinical decision process. To date, masochism seems to be the most common paraphilic interest among women^11,35–37^, which may have also affected the views of MHP. Of note is the fact that sexual masochism was also the only paraphilic behavior in the present study that did not involve a possibly non-consenting person, as is the case in exhibitionism, frotteurism, sexual sadism and pedophilia. Thus, even if paraphilias are considered to be typically male mental disorders, and male mental disorders seem to be characterized by externalizing symptoms, sexual masochism can be seen as the paraphilia with the most internalizing symptoms^28^. Interestingly, sexual sadism was the only paraphilia that was more pathologized by MHP with a psychoanalytic background compared to MHP with a cognitive-behavioral background. This is worth noting, as it may indicate that psychoanalytic MHP still assume more (presumably unconsciously) pathology in people with sadistic sexual behavior.

Paraphilias (particularly in women), apart from in forensic settings, have been neglected in terms of research. To date, neither has research on the actual prevalence rates been conducted, nor has the utility of the diagnostic criteria of paraphilic disorders been tested in DSM field trials^29^. This is striking, given the legal and social consequences of paraphilic disorders. Indeed, the few existing studies indicate that atypical interests and behaviors are common in both genders and challenge “…the idea that women’s sexual interests are necessarily centered on *normophilic* behaviors”^11,14,38^.

Overall, paraphilias were more stigmatized and less pathologized compared to the psychosis vignette. This result is not surprising because paraphilias are among the most stigmatized mental disorders^39^. Interestingly, the order of overall stigmatization in the present study can be understood as an expression of the forensic psychiatric relevance of disorders. Behaviors involving no bodily contact (hands-off behavior) seem to be less stigmatized then such potential hands-on behaviors as sadism or pedophilia. This was particularly true for the stigma-dimension “blame” (Table 2).

A limitation of the present study is that we did not assess all of the atypical sexual behaviors that are listed in DSM and ICD (e.g. fetishism or voyeurism). We cannot rule out that our stigma results are thus confounded by the fact that the atypical sexual behaviors surveyed either potentially include non-consenting individuals (sadism, pedophilia, exhibitionism, and frotteurism) and thus constitute crimes, or were arguably self-harming (masochism). Thus even if no stigma against atypical sexual behaviors existed, these behaviors might still be stigmatized for being crimes. Nevertheless, we were particularly interested in those behaviors that might be of legal importance, for example in criminal trials. In addition, always presenting the psychosis vignette first may have led to an order effect in the rating of subsequent disorders, an aspect which needs to be considered as a further limitation. The fact that 47% of the approached participants did not finish the study is a further methodological limitation. The present study included MHP from Germany, Austria and the German-speaking part of Switzerland, and we cannot rule out a selection bias. For example, those MHP who are less busy and are particularly interested in scientific experiments, and show more openness to sexuality, may have been more likely to complete the online questionnaire. While the present results may be representative of the Western world, given the important cultural impact on sexual norms, they cannot be generalized to other countries. In order to examine different cultural influences on the pathologization of atypical sexual behaviors, it may be fruitful in the future to compare MHP from culturally-diverse countries.

In conclusion, we have demonstrated for the first time a gender-bias in the perception and diagnosis of paraphilic disorders by male and female MHP. MHP are the most crucial group when it comes to differentiating between non-disordered and mentally disordered sexuality nowadays. Our data indicate that most atypical sexual behaviors are less pathologized in women. The underlying mechanisms and sociosexual norms leading to this bias should be addressed in future research because it has been shown that gender bias may have a serious impact on clinical and legal decisions^40^. Consequently, most ethical guidelines for MHP postulate a restriction of bias in clinical practice^41,42^. In future, it would also be interesting to explore whether or not gender influences other stigma facets, such as prognostic pessimism, which may influence therapeutic decisions.

Thus, although diagnostic criteria for paraphilic disorders exist, our study reveals that the pathologization of atypical sexual behavior still seems to be profoundly affected by nosologically irrelevant sexual norms. When confronted with atypical sexual expressions, MHP are therefore at risk of promoting gender stereotypes and sexual morality.

## Methods

### Recruitment procedure

MHP were recruited by means of an email invitation to complete an anonymous online survey concerning ‘diagnostic evaluation of sexual behavior’. Email invitations were sent to the chief physicians of psychiatric and psychosomatic hospitals in Germany, Austria and the German-speaking part of Switzerland. They were asked to distribute an invitation and a link to the study amongst their psychiatrists and clinical psychologists. At the beginning of the anonymous survey we fully explained the procedure, and participants had to give informed consent before starting the study. The local ethics committee (Psychotherapeutic Chamber Hamburg) approved the study protocol and all experiments were performed in accordance with relevant guidelines and regulations. In total, 971 individuals clicked on the survey link and 546 MHP (53%) completed it. The greatest number of drop-outs (81%) occurred before any question had been presented.

### Study design

Participants were randomly assigned to six case vignettes. The first vignette described an individual with psychotic symptoms followed by five vignettes describing subjects with atypical sexual interests or behaviors (exhibitionistic, frotteuristic, sexual sadistic, pedophilic, and sexual masochistic) and one vignette with non-paraphilic compulsive sexual behaviour, which will be reported elsewhere) (for detailed descriptions of the vignettes see Supplementary File 1). The vignettes gave detailed information about the person’s atypical sexual arousal pattern, which was manifested by sexual thoughts, fantasies, urges or behaviors. Moreover, vignettes in the fulfilled criteria condition included information about the duration criterion (over a period of at least 6 months) and acting out of the arousal pattern with individuals unwilling or unable to consent, or associated with impairments in functioning, according to DSM-5 criteria^17^. In contrast, in the ambiguous condition, no information about duration and consent or impairment in functioning was given. For each vignette, MHP were randomly assigned to one cell of a 2 “gender” (male/female) × 2 “sexual orientation” (homosexual/heterosexual) × 2 “diagnostic criteria” (fulfilled/ambiguous) factorial design. Participants were instructed to read the vignettes carefully and, after each vignette, were asked to rate their subjective estimation of the person’s mental health on a 6-point scale, and to indicate whether or not the person fulfilled the diagnostic criteria of a mental disorder according to DSM or ICD (Fig. 1). Qualitative data were collected in an open-ended text box asking participants to explain their answer. In addition, we adapted items used by Boysen and colleagues^28^ to measure the degree of stigma directed toward persons expressing paraphilic preferences. For each vignette, participants provided a stigma rating on a six-point Likert scale (strongly disagree - strongly agree) that included the following stigma dimensions: desire for social distance (“*I feel irritation towards Mr./Mrs. X*.”;, “*If I were a landlord, I probably would rent an apartment to Mr./Mrs. X*.”); perceived dangerousness (“*I would feel threatened by Mr./Mrs. X*.”; “*Mr./Mrs. X is dangerous*.”); blame (“*I think it is the fault of Mr./Mrs. X that he/she is in that condition*.”; “*Mr./Mrs. X should be punished*.”; “*Mr./Mrs. X*. *could control his/her behavior if she/he wanted to*”). In addition, the seven stigma items were combined to form a single stigmatization index. Moreover, all participants provided an opinion if the symptom was predominantly caused by biological or psychological factors by moving a slider between biological (0) and psychological (100) etiology (“*Please indicate if the fantasies/behavior of the person is “caused” by biological or psychological factors?”*). Additionally, in order to control for potential confounding variables, four items measuring the MHP attitudes were assessed on a six-point Likert scale (“*I am religious*”, “*I perceive myself as a member of a minority group*.”, “*Sexual behavior of men and women strongly differ*.” and “*I am politically*…”(1 = liberal to 6 = conservative).

### Statistical analyses

Statistical analyses were carried out using IBM SPSS Statistics 22.0 (IBM Corp., Armonk, NY). Data are reported as means ± S.E.M. A three-factorial analysis of variance (ANOVA), followed by Bonferroni’s post-hoc analysis, were carried out to study the effect of vignette version, gender and sexual orientation on the estimation of psychopathology and stigmatization. A chi-square test was performed to compare the percentage of MHP diagnosing a mental disorder, and for the quantification of qualitative data. Qualitative data were analyzed using content analysis^43,44^. They were first rated by a coder in order to determine categories. All data were subsequently coded by two independent raters, who were trained by two of the authors (VK & JF). Cohen’s kappa was calculated to compare the inter-rater reliability, and is reported in Supplemental Table 1. The kappa value can be interpreted according to Altman (1991). It was very good for 52 categories (0.81–1) and good for 47 categories (0.61–0.80). Only 4 categories had a moderate (<0.61) inter-rater reliability. Significance was evaluated at a probability of 5% or less (<0.05).

### Data availability statement

We confirm that all relevant data are available from the authors.

## Electronic supplementary material

Supplemental File 1
