# Author Correction: Gender bias in clinicians’ pathologization of atypical sexuality: a randomized controlled trial with mental health professionals

**DOI:** 10.1038/s41598-018-24181-w

**Published:** 2018-04-16

**Authors:** Johannes Fuss, Peer Briken, Verena Klein

**Affiliations:** Institute for Sex Research and Forensic Psychiatry, Center of Psychosocial Medicine, University Medical Center Hamburg-Eppendorf, Hamburg, Germany

Correction to: *Scientific Reports* 10.1038/s41598-018-22108-z, published online 27 February 2018

The original version of this Article contained an error in the Abstract.

“Female subjects were significantly less pathologized and overall more stigmatized in terms of exhibitionistic, frotteuristic, sexual sadistic and pedophilic behavior.”

now reads:

“Female subjects were significantly less pathologized and overall less stigmatized in terms of exhibitionistic, frotteuristic, sexual sadistic and pedophilic behavior.”

This has now been corrected in the PDF and HTML versions of the Article.

