# Supplementary material for: Gender bias in clinicians’ pathologization of atypical sexuality: a randomized controlled trial with mental health professionals
Source: Sci Rep. 2018 Feb 27;8:3715. doi: 10.1038/s41598-018-22108-z (PMC5829224; doi:10.1038/s41598-018-22108-z)
Supplement: Supplementary file 1 — Supplemental File 1 [file 41598_2018_22108_MOESM1_ESM.pdf]

Supplemental information for:

Gender bias in clinicians' pathologization of atypical sexuality: a randomized controlled trial with mental health professionals

Johannes Fuss*, Peer Briken, Verena Klein*

Institute for Sex Research and Forensic Psychiatry, Center of Psychosocial Medicine, University Medical Center Hamburg-Eppendorf, Germany

* Corresponding authors:

Institute for Sex Research and Forensic Psychiatry

Center of Psychosocial Medicine

University Medical Center Hamburg-Eppendorf

Martinistraße 52

20246 Hamburg

Germany

Phone: +49-40-7410-52225

Fax: +49-40-7410- 56406

E-Mail: v.klein@uke.de & jo.fuss@uke.de

Supplemental table 1: Content analyses of mental health professionals‘explanations

**PSYCHOSIS**	Kappa	Vignette			
		Ambiguous		Full criteria	
		Male (n = 39)	Female (n = 38)	Male (n = 33)	Female (n = 42)
Arguments for a diagnosis					
Any pro argument	0.964	35 (0.90)	35 (0.92)	29 (0.88)	38 (0.90)
Criteria fulfilled	0,874	4 (0.10)	4 (0.11)	7 (0.21)	8 (0.19)
Time criteria	1	3 (0.08)	1 (0.03)	4 (0.12)	3 (0.07)
Psychosis	0.759	12 (0.31)	10 (0.26)	5 (0.15)	10 (0.24)
Hallucinations	0.908	13 (0.33)	16 (0.42)	11 (0.33)	10 (0.24)
Delusions	0.934	18 (0.46)	21 (0.55)	16 (0.49)	17 (0.40)
Thought disorder	0.910	3 (0.08)	1 (0.03)	1 (0.03)	6 (0.14)
“Ich-Störung”	0.907	11 (0.28)	6 (0.16)	6 (0.18)	4 (0.10)
Social withdrawal	0.978	5 (0.13)	11 (0.29)	8 (0.24)	5 (0.12)
Decompensation	0.859	3 (0.08)	4 (0.11)	3 (0.09)	1 (0.02)
Distress	1	2 (0.05)	1 (0.03)	1 (0.03)	1 (0.02)
Suicide attempt	0.973	10 (0.26)	3 (0.08)	3 (0.09)	6 (0.14)
Arguments against a diagnosis					
Any contra argument	0.824	10 (0.26)	7 (0.18)	4 (0.12)	4 (0.10)
Criteria not fulfilled	0.689	5 (0.13)	2 (0.05)	2 (0.06)	1 (0.02)
Time criteria not fulfilled	0.743	1 (0.02)	1 (0.03)	1 (0.03)	1 (0.02)
Pro and contra arguments					
Balance/weigh	0.886	5 (0.13)	6 (0.16)	1 (0.03)	1 (0.02)
No effect reasons					
Not enough information	0.661	1 (0.03)	1 (0.03)	1 (0.02)	0

EXHIBITIONISM	Kappa	Vignette			
		Ambiguous		Full criteria	
		Male (n = 41)	Female (n = 37)	Male (n = 22)	Female (n = 37)
Arguments for a diagnosis					
Any pro argument	0.726	26 (0.63)	9 (0.24)	17 (0.77)	23 (0.62)
Criteria fulfilled	0.926	15 (0.37)	3 (0.08)	9 (0.40)	10 (0.27)
Exhibitionistic sexual interest	0.951	12 (0.29)	2 (0.05)	6 (0.27)	6 (0.16)
Deviant sexual interests	0.802	3 (0.07)	0	1 (0.05)	7 (0.19)
Distress	0.885	2 (0.05)	1 (0.03)	1 (0.05)	1 (0.03)
Threat to others/conflicts	0.795	3 (0.07)	0	6 (0.27)	5 (0.14)
Abnormal sexual interest	0.758	3 (0.07)	2 (0.05)	0	1 (0.03)
Acting out	0.964	3 (0.07)	4 (0.10)	3 (0.13)	5 (0.14)
Public	0.762	2 (0.05)	2 (0.05)	1 (0.05)	3. (0.08)
Impulse control deficit	1.00	1 (0.02)	1 (0.03)	1 (0.05)	1 (0.03)
Has a personality disorder	1.00	0	1 (0.03)	0	3. (0.08)
Arguments against a diagnosis					
Any contra argument	0.604	17 (0.42)	22 (0.60)	7 (0.32)	10 (0.27)
No distress	0.872	9 (0.22)	8 (0.22)	3 (0.14)	5 (0.14)
Criteria not fullfilled	0.562	1 (0.02)	2 (0.05)	0	0
No threat to others	0.761	5 (0.12)	7 (0.19)	0	3 (0.08)
Behavioral control	0.828	6 (0.15)	7 (0.19)	0	1 (0.03)
Sexual interest is not a mental disorder	0.858	3 (0.07)	7 (0.19)	3 (0.14)	3 (0.08)
No public	0.653	3 (0.07)	5 (0.14)	0	0
Gender	1.00	0	0	0	4 (0.10)
Pro and contra arguments					
Balance/weigh	0.658	13 (0.32)	8 (0.22)	4 (0.18)	10 (0.27)
No effect reasons					
Not enough information	0.784	6 (0.14)	7 (0.19)	1 (0.05)	6 (0.16)

FROTTEURISM	Kappa	Vignette			
		Ambiguous		Full criteria	
		Male (n = 33)	Female (n = 23)	Male (n = 21)	Female (n = 30)
Arguments for a diagnosis					
Any pro argument	0.862	19 (0.58)	12 (0.52)	16 (0.76)	20 (0.67)
Criteria fulfilled	0.858	12 (0.36)	7 (0.30)	8 (0.38)	9 (0.30)
Frotteuristic sexual interest	0.976	12 (0.36)	5 (0.22)	3 (0.14)	8 (0.27)
Acting out	0.795	1 (0.03)	1 (0.04)	0	1 (0.03)
No consent	0.797	8 (0.24)	4 (0.17)	9 (0.43)	10 (0.33)
Abnormal sexual interest	0.942	3 (0.09)	1 (0.04)	5 (0.24)	1 (0.03)
Impulse control deficit	1.00	1 (0.03)	0	2 (0.10)	4 (0.13)
Arguments against a diagnosis					
Any contra argument	0.634	11 (0.33)	6 (0.26)	3 (0.14)	4 (0.13)
No distress	0.825	8 (0.24)	3 (0.13)	0	3 (0.10)
No threat to others	0.791	4 (0.12)	2 (0.09)	0	0
Sexual interest is not a mental disorder	0.827	2 (0.06)	3 (0.13)	4 (0.19)	1 (0.03)
Gender	1.00	0	1 (0.04)	0	1 (0.03)
Pro and contra arguments					
Balance/weigh	0.755	8 (0.24)	3 (0.13)	5 (0.24)	6 (0.20)
No effect reasons					
Not enough information	0.841	3 (0.09)	1 (0.04)	1 (0.10)	4 (0.13)

**SEXUAL SADISM**	Kappa	Vignette			
		Ambiguous		Full criteria	
		Male (n = 28)	Female (n = 39)	Male (n = 32)	Female (n = 33)
Arguments for a diagnosis					
Any pro argument	0.907	15 (0.54)	17 (0.44)	22 (0.69)	23 (0.70)
Criteria fulfilled	0.697	8 (0.27)	4 (0.10)	9 (0.28)	8 (0.24)
Sadistic sexual interest	0.776	6 (0.21)	5 (0.13)	8 (0.25)	11 (0.33)
Threat to others	0.799	7 (0.25)	8 (0.21)	7 (0.22)	8 (0.24)
Exclusive sexual interest	0.929	4 (0.14)	1 (0.03)	1 (0.03)	2 (0.06)
No consent	0.747	3 (0.11)	4 (0.11)	14 (0.44)	12 (0.36)
Abnormal	0.585	1 (0.04)	2 (0.05)	1 (0.03)	0
Impulse control deficit	1.00	0	2 (0.05)	2 (0.06	1 (0.03)
Has a personality disorder	0.885	1 (0.04)	0	3 (0.09))	0
Arguments against a diagnosis					
Any contra argument	0.662	10 (0.36)	18 (0.46)	7 (0.22)	7 (0.22)
No distress	0.788	1 (0.04)	4 (0.10)	0	2 (0.06)
Consent	0.802	9 (0.32)	14 (0.36)	5 (0.16)	4 (0.12)
Sexual interest is not a mental disorder	0.638	0	5 (0.13)	5 (0.16)	2 (0.06)
Gender	1.00	0	1 (0.03)	0	0
Pro and contra arguments					
Balance/weigh	0.860	5 (0.18)	10 (0.26)	9 (0.28)	2 (0.06)
No effect reasons					
Not enough information	0.949	4 (0.14)	9 (0.23)	6 (0.19)	5 (0.15)

**PEDOPHILIA**	Kappa	Vignette			
		Ambiguous		Full criteria	
		Male (n = 30)	Female (n = 25)	Male (n = 19)	Female (n = 24)
Arguments for a diagnosis					
Any pro argument	0.617	22 (0.73)	15 (0.60)	19 (1)	22 (0.92)
Criteria fulfilled	0.752	5 (0.17)	5 (0.20)	1 (0.05)	6 (0.25)
Pedophilic interest	0.551	10 (0.33)	9 (0.36)	11 (0.58)	14 (0.58)
Distress	0.638	6 (0.20)	3 (0.12)	2 (0.11)	1 (0.04)
Sexual interest is unchangeable	0.753	1 (0.03)	1 (0.04)	1 (0.05)	3 (0.13)
Treatment might help	0.584	4 (0.13)	3 (0.12)	3 (0.16)	3 (0.13)
Threat to others	0.745	6 (0.20)	1 (0.04)	7 (0.37)	3 (0.13)
Exclusive pedophilic interest	0.709	3 (0.10)	0	3 (0.16)	3 (0.13)
Acting out	0.712	0	2 (0.08)	0	5 (0.21)
Arguments against a diagnosis					
Any contra argument	0.763	15 (0.50)	17 (0.68)	3 (0.16)	7 (0.29)
Criteria not fulfilled	0.662	1 (0.03)	0	0	1 (0.04)
No threat to others	0.711	0	4 (0.16)	0	1 (0.04)
No distress	0.643	3 (0.10)	6 (0.24)	0	1 (0.04)
Behavioral control	0.792	10 (0.33)	13 (0.52)	2 (0.11)	4 (0.17)
Sexual interest in no mental disorder	0.712	3 (0.10)	0	2 (0.11)	2 (0.08)
No exclusivity	1	4 (0.13)	1 (0.04)	0	0
Gender	0.852	0	3 (0.12)	0	1 (0.04)
Pro and contra arguments					
Balance/weigh	0.739	11 (0.37)	4 (0.16)	3 (0.16)	1 (0.04)
No effect reasons					
Not enough information	0.852	9 (0.30)	2 (0.08)	0	1 (0.04)

Supplemental Figure 1: Effect of criteria condition on psychopathology score

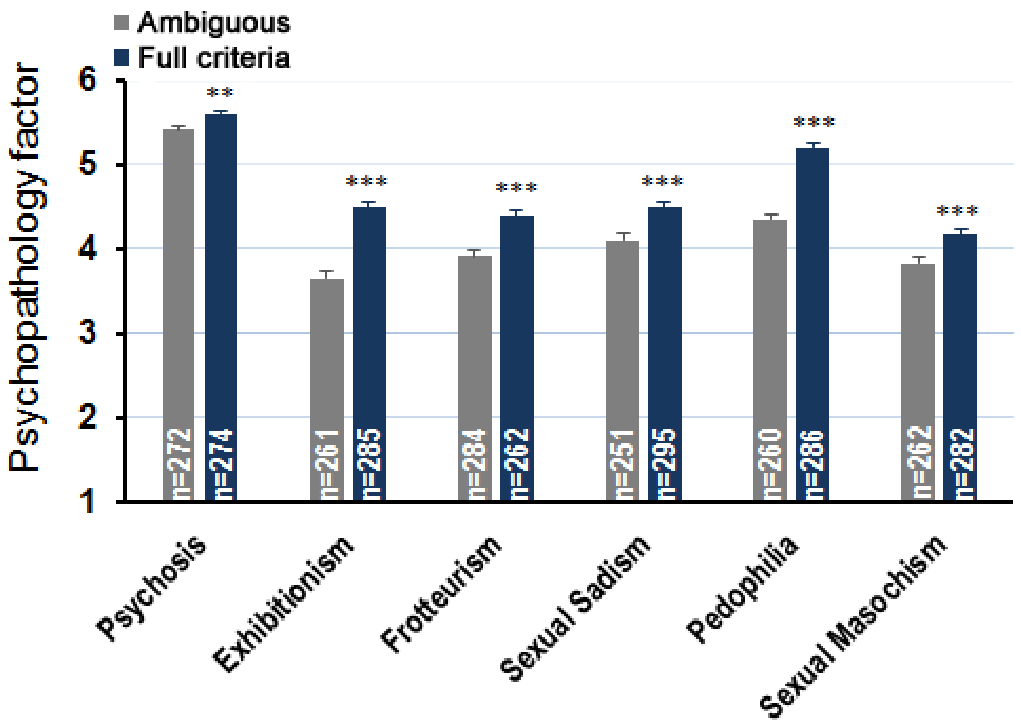

Supplemental Figure 2: Effect of sexual orientation on psychopathology score

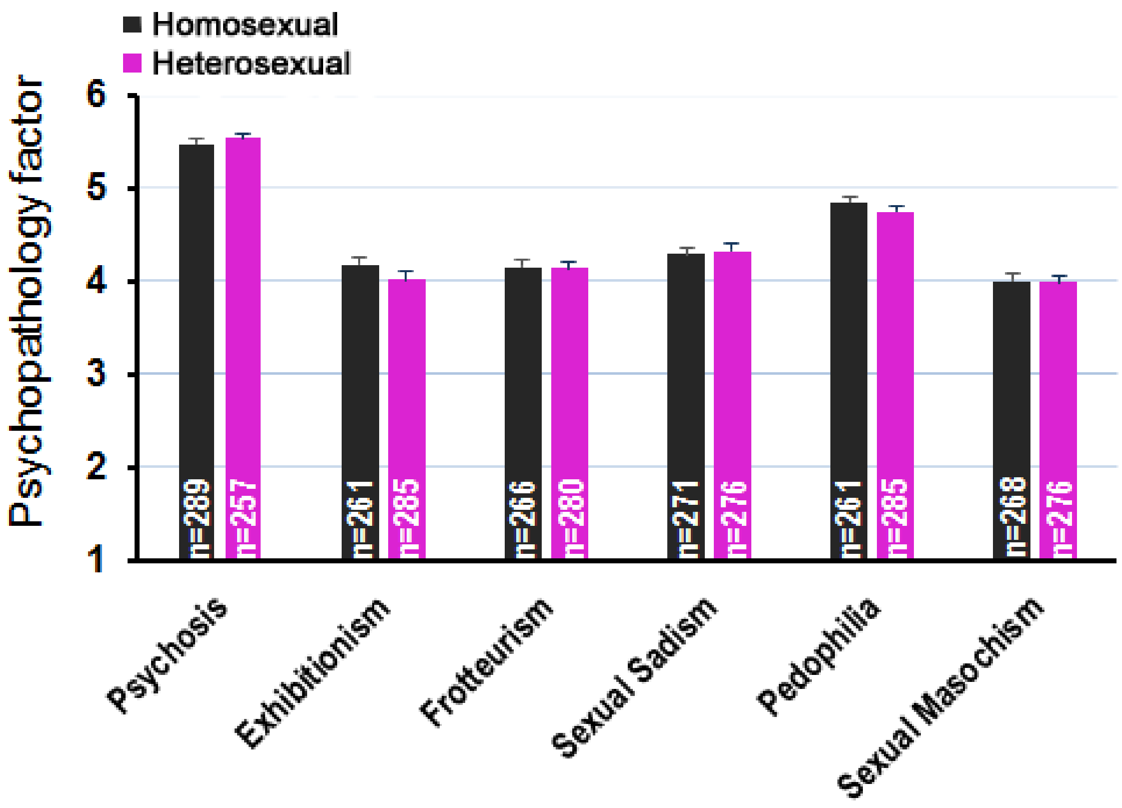

Supplemental Information: Vignettes

For all vignettes, red words were altered for sexual orientation (e.g., girlfriend instead of boyfriend) and black words for gender (e.g., Mrs instead of Mr.).

Psychosis Vignette

Homosexual male

Full criteria condition

German original: Herr M. hört seit einem Jahr die Stimmen seiner Nachbarn, die sein Verhalten kommentieren. Er ist sich sicher, dass die Nachbarn ihn auch überwachen und kontrollieren, da diese immer zur Tür kommen, wenn er seine Wohnung verlässt. Er beschreibt, dass die Nachbarn Spezialkräfte haben, sie können die Gedanken anderer Menschen lesen und steuern. Herr M. hat sein Studium abgebrochen, da er sich darauf im Moment nicht konzentrieren kann. Er wohnt allein, nachdem sich sein Freund vor 1.5 Jahren von ihm trennte. Vor einem Jahr hat Herr M. einen Suizidversuch begangen.

English translation: For the last year **Mr**. M has been hearing the voices of **his** neighbors commenting on **his** behavior. **He** is convinced they are monitoring and controlling **him** as they always come to the front door when **he** is leaving **his** apartment. **He** reports that **his** neighbors have supernatural abilities: they are able to read and manipulate people's thoughts. **Mr**. M quit university as **he** was no longer able to concentrate. **He** was left by **his** **boyfriend** 1.5 years ago; since then **he** has been living alone. A year ago **Mr**. M attempted suicide.

Ambiguous condition

German original: Herr M. hört in seiner Wohnung manchmal die Stimmen seiner Nachbarn, die sein Verhalten kommentieren. Er hat das Gefühl, dass die Nachbarn ihn auch überwachen und kontrollieren, da diese häufig zur Türe kommen, wenn er seine Wohnung verlässt. Manchmal hat er das Gefühl, die Nachbarn haben Spezialkräfte, sie können z.B. die Gedanken anderer Menschen lesen und steuern. Herr M. hat sein Studium abgebrochen. Er wohnt allein, nachdem sich sein Freund vor 1.5 Jahren von ihm trennte. Vor einem Jahr hat Herr M. einen Suizidversuch begangen.

English translation: For the last year **Mr**. M has occasionally been hearing the voices of **his** neighbors commenting on **his** behavior. **He** believes they are monitoring and controlling **him** as they often come to the front door when **he** is leaving **his** apartment. **He** sometimes feels that **his** neighbors have supernatural abilities, such as the power to read and manipulate people's thoughts. **Mr**. M quit university. **He** was left by **his** **boyfriend** 1.5 years ago; since then **he** has been living alone. A year ago **Mr**. M attempted suicide.

Exhibitionism Vignette

Heterosexual male

Full criteria condition

German original: Herr L. ist seit seiner Jugend sexuell erregt, wenn er sich nackt in der Öffentlichkeit zeigt. Von unbekannten Frauen betrachtet zu werden, erregt ihn am meisten. Er befriedigt sich regelmäßig selbst zu der Phantasie, dass die Frauen Lust bekommen mit ihm Sex zu haben, wenn sie ihn ansehen. In seiner Wohnung im 2. Stock einer belebten Straße befriedigt er sich häufig selbst. Dabei steht er gut sichtbar am Fenster bei geöffneten Vorhängen. Dieses Verhalten erregt ihn sehr.

English translation: Since **his** adolescence, **Mr**. L derives sexual pleasure from exposing **himself** nude in public. **He** is most intensely aroused when being observed by **female** strangers. **He** regularly masturbates to the sexual fantasy that these **women**, when watching **him**, become turned on and want to have sex with **him**. **His** apartment is on the second floor facing a busy street. **He** often masturbates visibly at the window with open curtains; **he** finds this behavior deeply arousing.

Ambiguous condition

German original: Herr L. ist sexuell erregt, wenn er sich nackt in der Öffentlichkeit zeigt. Von unbekannten Frauen betrachtet zu werden, erregt ihn am meisten. Er befriedigt sich regelmäßig selbst zu der Phantasie, dass die Frauen Lust bekommen mit ihm Sex zu haben, wenn sie ihn ansehen. In seiner Wohnung im 2. Stock einer belebten Straße befriedigt er sich gelegentlich selbst. Dabei steht er hinter dem Vorhang versteckt am Fenster. Dieses Verhalten erregt ihn sehr.

English translation: **Mr**. L derives sexual pleasure from exposing **himself** nude in public. **He** is most intensely aroused when being observed by **female** strangers. **He** regularly masturbates to the sexual fantasy that these **women**, when watching **him**, become turned on and want to have sex with **him**. **His** apartment is on the second floor facing a busy road. **He** occasionally masturbates at the window hidden behind closed curtains; **he** finds this behavior deeply arousing.

Frotteurism Vignette

<u>Homosexual female</u>

Full criteria condition

German original: Frau G. ist seit dem frühen Erwachsenenalter sexuell erregt, wenn sie sich im Gedränge an fremden Frauen reibt. Aufgrund dieser Vorliebe reibt sie sich gelegentlich auch an ihrer Partnerin, um ihre Klitoris zu stimulieren. Frau G. ist in der Öffentlichkeit auch schon zum Höhepunkt gekommen, weil sie sich im Gedränge an fremde Frauen gepresst hat. Die Vorstellung, dass die Frau, an der sie sich reibt, nichts von ihrer Erregung weiß und durch ihr Verhalten irritiert wird, hat einen zusätzlichen Reiz. Frau G. wurde für ihr Verhalten bereits in der Öffentlichkeit beschimpft.

English translation: Since early adulthood, **Mrs**. G is sexually aroused when rubbing and pressing **herself** against **female** strangers in crowds. Consequently, **she** sometimes rubs **herself** against **her** **girlfriend** to stimulate **her** clitoris. **Mrs**. G has even reached orgasm by pressing and rubbing **herself** against **female** strangers in a crowd. The thought that these **women** don’t know of **her** stimulation and may be irritated by **her** behavior provides additional arousal. **Mrs**. G has been reprimanded in public for **her** behavior.

Ambiguous condition

German original: Frau G. findet es ist sexuell erregend, wenn sie sich im Gedränge an fremden Frauen reibt. Aufgrund dieser Vorliebe reibt sie sich gelegentlich auch an ihrer Partnerin, um ihre Klitoris zu stimulieren. Frau G. ist in der Öffentlichkeit auch schon zum Höhepunkt gekommen, weil durch enges Gedränge fremde Frauen an sie gepresst wurden. Die Vorstellung, dass die Frau, an die sie gedrückt wird, nichts von ihrer Erregung weiß und durch ihr Verhalten irritiert werden könnte, hat einen zusätzlichen Reiz.

English translation: **Mrs**. G is sexually aroused when rubbing and pressing **herself** against **female** strangers in crowds. Consequently, **she** sometimes rubs **herself** against **her** **girlfriend** to stimulate **her** clitoris. **Mrs**. G has even reached orgasm when being pressed against **female** strangers in a crowd. The thought that these **women** don’t know of **her** stimulation and may be irritated by **her** behavior provides additional arousal.

Sexual Sadism Vignette

<u>Heterosexual female</u>

Full criteria condition

German original: Seit vielen Jahren ist Frau B. bei sexuellen Kontakten dominant. Besonders das Demütigen ihrer Partner erregt sie. Wenn sie merkt, dass diese Gefallen daran haben, ohrfeigt sie sie auch und spuckt sie an. Vor kurzem kam es zu Geschlechtsverkehr mit ihrem derzeitigen Partner Christian, sie fesselte und knebelte ihn. Es erregte sie zu wissen, dass Christian ihr hilflos ausgeliefert ist. Dann verbrannte sie ihn mit brennenden Zigaretten im Brustbereich, obwohl Christian versuchte sich zu wehren.

English translation: **Mrs**. B has been dominant in sexual encounters for many years. **She** is particularly aroused when humiliating **her** partners. If **she** perceives that they enjoy the humiliation **she** also slaps them in the face and spits at them. Recently **she** hogtied and gagged **her** present **boyfriend Christian** during sexual intercourse. It aroused **her** to know that **Christian** was helplessly at **her** mercy. **She** then burnt **his** chest using lit cigarettes, even though **Christian** was attempting to defend **himself**.

Ambiguous condition

German original: Frau B. ist bei sexuellen Kontakten dominant. Besonders das Demütigen ihrer Partner erregt sie. Wenn sie merkt, dass diese Gefallen daran haben, ohrfeigt sie sie auch und spuckt sie an. Vor kurzem kam es zu Geschlechtsverkehr mit ihrem derzeitigen Partner Christian, sie fesselte und knebelte ihn. Es erregte sie zu wissen, dass Christian ihr hilflos ausgeliefert ist. Dann verbrannte sie ihn mit brennenden Zigaretten im Brustbereich.

English translation: **Mrs**. B is dominant in sexual encounters. **She** is particularly aroused when humiliating **her** partners. If **she** perceives that they enjoy the humiliation **she** also slaps them in the face and spits at them. Recently **she** hogtied and gagged **her** present **boyfriend Christian** during sexual intercourse. It aroused **her** to know that **Christian** was helplessly at **her** mercy. **She** then burnt **his** chest using lit cigarettes.

Pedophilia Vignette

Homosexual female

Full criteria condition

German original: Frau Z. berichtet seit dem frühen Erwachsenenalter sexuelle Phantasien, die sich auf Mädchen im Alter von acht Jahren beziehen. Regelmäßig schaut sie sich Darstellungen von Mädchen in Unterwäsche an, stellt sich Geschlechtsverkehr mit diesen vor und befriedigt sich dabei selbst. Sie hat kein sexuelles Interesse an erwachsenen Frauen. Ab und an geht sie in der Nähe einer Schule spazieren, um Kinder beim Spielen zu beobachten. Sie ist von dem Anblick der Mädchen sexuell sehr erregt und spürt dabei häufig ein Feuchtwerden und Pulsieren im Schritt. Sie möchte gerne eine sexuelle Beziehung zu einem 8-jährigen Mädchen, hat sich aber bisher nicht getraut sie anzusprechen.

English translation: **Mrs**. Z describes having sexual fantasies involving eight-year-old **girls** since early adulthood. Whilst masturbating **she** regularly views pictures of **girls** in underwear and fantasizes about having sex with them. **She** is not sexually interested in adult **women**. From time to time **she** goes for a stroll near a school to observe the children as they play. Merely watching the young **girls** is intensely arousing; **she** often becomes **wet** and feels **strong pulsations of excitement**. **She** would like to begin sexual relations with an eight-year-old **girl**, but has not yet dared speak to **her**.

Ambiguous condition

German original: Frau Z. berichtet sexuelle Phantasien, die sich auf Mädchen im Alter von acht Jahren beziehen. Manchmal schaut sie sich Darstellungen von Mädchen in Unterwäsche an, stellt sich Geschlechtsverkehr mit diesen vor und befriedigt sich dabei selbst. Sie hat auch sexuelles Interesse an erwachsenen Frauen. Als sie kürzlich in der Nähe einer Schule spazieren ging, beobachtete sie Kinder beim Spielen. Beim Anblick der Mädchen war sie sexuell sehr erregt und spürte dabei ein Feuchtwerden und Pulsieren im Schritt. Sie möchte keine sexuelle Beziehung zu einem Kind eingehen.

English translation: **Mrs**. Z describes having sexual fantasies involving eight-year-old **girls**. Whilst masturbating **she** occasionally views pictures of **girls** in underwear and fantasizes about having sex with them. **She** is also sexually interested in adult **women**. During a recent stroll **she** happened to pass near a school and observed the children play. Merely watching the young **girls** was intensely arousing; **she** became **wet** and felt **strong pulsations of excitement**. **She** does not wish to have sexual relations with a child.

Masochism vignette

Homosexual male

Full criteria condition

German original: Herr N. berichtet, dass er seit vielen Jahren sexuell erregt wird, wenn ihn sein Lebenspartner demütigt. Dieser beschimpft Herr N., fesselt ihn ans Bett und schlägt ihn. Besonders das Gefühl des ihm Ausgeliefertseins und die ihm zugefügten Schmerzen erlebt Herr N. als sexuell erregend und lustvoll. Die sexuellen Begegnungen gehen häufig mit Prellungen, Blutergüssen und Kratzwunden einher. Herr N. musste aufgrund der entstandenen Verletzungen wiederholt ins Krankenhaus. Herr N. berichtet sexuelle Phantasien, in

English translation: For many years **Mr**. N has derived sexual pleasure from being humiliated by **his** **boyfriend**. Said **boyfriend** swears at **him**, hogties **him** to the bed and beats **him**. Being at **his** **boyfriend**'s mercy and feeling the inflicted pain is particularly arousing and stimulating. Sexual encounters often produce injuries such as bruises, hematomas and scratch marks. **Mr**. N has repeatedly required medical attention for injuries sustained during sex. **Mr**. N describes sexual fantasies in which **he** is overpowered by a **man** and is forced to submit to **his** sexual demands.

Ambiguous condition

German original: Herr N. berichtet, dass er sexuell erregt wird, wenn ihn sein Lebenspartner demütigt. Dieser beschimpft Herr N., fesselt ihn ans Bett und schlägt ihn. Besonders das Gefühl des ihm Ausgeliefertseins und die ihm zugefügten Schmerzen erlebt Herr N. als sexuell erregend und lustvoll. Die sexuellen Begegnungen gehen manchmal mit Prellungen, Blutergüssen und Kratzwunden einher. Herr N. berichtet sexuelle Phantasien, in denen er von einem Mann überwältigt wird und sich dessen sexuellen Wünschen fügen muss.

English translation: **Mr**. N derives sexual pleasure from being humiliated by **his** **boyfriend**. Said **boyfriend** swears at **him**, hogties **him** to the bed and beats **him**. Being at **his** **boyfriend's** mercy and feeling the inflicted pain is particularly arousing and stimulating. Sexual encounters sometimes produce injuries such as bruises, hematomas and scratch marks. **Mr**. N describes sexual fantasies in which **he** is overpowered by a **man** and is forced to submit to **his** sexual demands.
